# qPCR as a Selective Tool for Cytogenetics

**DOI:** 10.3390/plants12010080

**Published:** 2022-12-23

**Authors:** Mikhail G. Divashuk, Ekaterina A. Nikitina, Victoria M. Sokolova, Anna I. Yurkina, Alina A. Kocheshkova, Olga V. Razumova, Gennady I. Karlov, Pavel Yu. Kroupin

**Affiliations:** All-Russia Research Institute of Agricultural Biotechnology, 127550 Moscow, Russia

**Keywords:** fluorescent in situ hybridization, tandem satellite repeats, wheat, wheatgrass, sea buckthorn, DNA repeats, whole genome sequencing, copy number

## Abstract

qPCR is widely used in quantitative studies of plant genomes and transcriptomes. In this article, this method is considered as an auxiliary step in the preparation and selection of markers for FISH analysis. Several cases from the authors’ research on populations of the same species were reviewed, and a comparison of the closely related species, as well as the adaptation of the markers, based on satellite tandem repeats (TRs) using quantitative qPCR data was conducted. In the selected cases, TRs with contrast abundance were identified in the cases of the *Dasypyrum, Thinopyrum* and *Aegilops* species, and the transfer of TRs between the wheat and related species was demonstrated. TRs with intraspecific copy number variation were revealed in *Thinopyrum ponticum* and wheat-wheatgrass partial amphidiploids, and the TR showing predominant hybridization to the sea buckthorn Y chromosome was identified. Additionally, problems such as the absence of a reference gene for qPCR, and low-efficiency and self-complementary primers, were illustrated. In the cases considered here, the qPCR results clearly show high correlation with the subsequent results of the FISH analysis, which confirms the value of this method for cytogenetic studies.

## 1. Introduction

Fluorescence in situ hybridization (FISH) is a powerful and popular tool for the cytogenetic study of plant genomes. FISH analysis can be used for chromosome identification, the mapping of alien introgressions, the study of phylogenetic relationships, and genome evolution [1,2,3,4]. One of the routine tasks of FISH analysis is the development of cytogenetic chromosome markers for fundamental and applied research. Repetitive DNA sequences, particularly satellite tandem repeats (TRs), are a frequent target for marker design. A typical TR monomer is composed of small DNA fragments with lengths of 50–500 nt. In total, a repeat of one type can reach a length of more than 10,000 nt. As the resolution of FISH is 10,000 nt or more, TRs are convenient for the development of cytogenetic markers. The TR monomer, despite the presence of conserved parts of the sequence, can be polymorphic, which creates further scope for the study of speciation and the relationships between closely related species. Information on the number of copies of the TR monomer and the stability of this parameter in a species is also valuable for researchers [5,6,7,8].

FISH analysis consists of several stages, some of which are time- and labor-intensive. For its optimization, it may be necessary to introduce steps including the selection of promising repeats, bioinformatic sequence analysis, and comparisons with previously published repeats. Laboratory confirmation of the presence of the repeat is most often performed by PCR, but this does not provide information on the quantitative expansion of the TR in the genome under investigation.

Southern and dot blot hybridization, as conventional but time- and labor-intensive methods for copy number estimation, have gradually been replaced by qPCR, which enables the rapid, high-quality, and low-cost analysis of the copy number of a given sequence [9,10,11]. Moreover, in the estimation of the repeated DNA copy number, qPCR was shown to be more precise than dot blot hybridization due to its higher stringency [12]. The qPCR method has been widely used to assess the copy number of different types of repeated genome sequences, including transposable elements and satellite repeats, in various biological objects [13,14,15,16,17,18,19].

**Figure 1:**
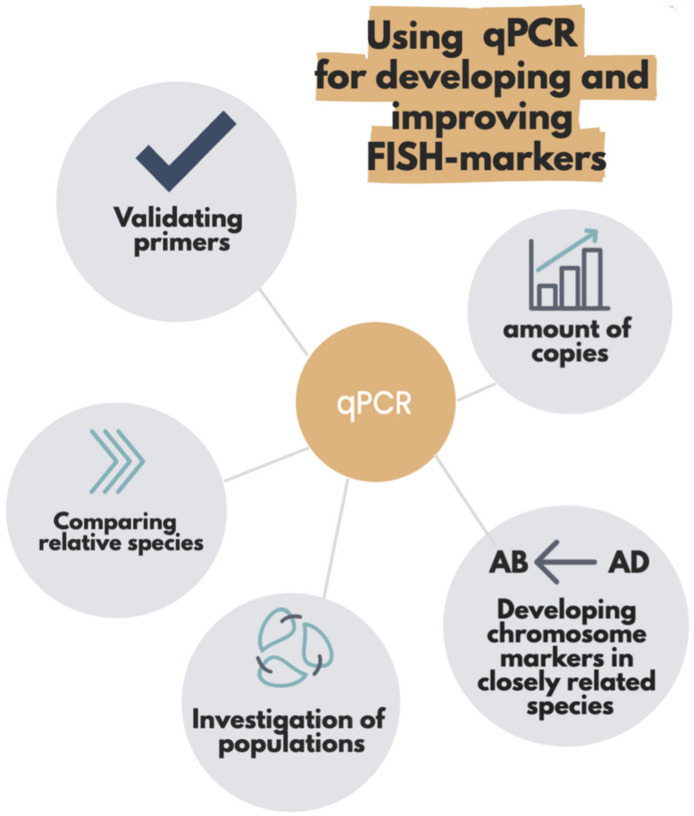
Methods of qPCR applied in different cases, including the development of FISH markers.

Through qPCR, a researcher can estimate the copy number of a particular TR in the genome and its variability among the genomes, thus enabling its initial comparative quantitative assessment before its conversion it into a chromosome marker (Figure 1).

Therefore, the application of qPCR as a preliminary and supplementary step in cytogenetic studies helps researchers to plan further experiments more efficiently [12,20,21,22,23,24,25]. Here, cases of successful qPCR application in cytogenetic studies based on various projects conducted by the research team are discussed, providing examples of problematic and ambiguous cases and the ways in which they were resolved.

## 2. Results

### 2.1. The Study of Closely Related Species

Often, the task of researchers is to compare the copy numbers of the same tandem repeat between two or more species of the same genus or between representatives of different genera. Such comparative studies demonstrate similarities and differences between the studied groups and are widely used in phylogenetic and evolutionary studies.

#### 2.1.1. pHv-961

**Figure 2:**
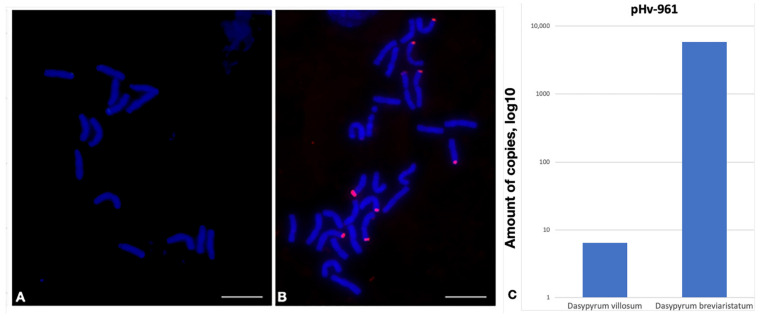
Localization of pHv-961 tandem repeat on the metaphase chromosomes of (**A**) *Dasypyrum villosum* PI 21717 and (**B**) *Dasypyrum breviaristatum* PI 516547 using fluorescence in situ hybridization. Red signals indicate the chromosomal localization of the pHv-961 tandem repeat. The bar indicates 10 µm. (**C**) Histogram of the copy number of the tandem repeat pHv-961 in *Dasypyrum villosum* PI 21717 and *Dasypyrum breviaristatum* PI 516547.

Repeat pHv-961, identified in the *Hordeum vulgare* genome (2*n* = 14, HH) [26], was used in this study to compare the TR abundance and chromosome positions of two closely related species with different ploidy levels, namely *Dasypyrum villosum* (2*n* = 14, VV) and *Dasypyrum breviaristatum* (2*n* = 28, VVVbVb), which have a similar common genome V [27]. The relative copy number of pHv-961 differed by approximately 1000 between the two closely related accessions. FISH confirmed that the difference in the copy number is clear, with bright signals visible on the *D. breviaristatum* chromosomes, while they were absent in *D. villosum* (Figure 2).

The significant difference in the copy number cannot be explained by the difference in the ploidy levels of the species. Judging by the fact that there are no signals in the chromosomes of the V genome of *D. villosum* and that they are observed on less than 14 chromosomes in *D. breviaristatum*, it can be assumed that the repeat specifically hybridizes to the chromosomes of the Vb subgenome of *D. breviaristatum*. Thus, qPCR identified a significant difference in the copy number of the pHv-961 repeat. Therefore, it was selected for further FISH experiments. Based on the results, its participation in the polyploidization of *D. breviaristatum* can be suggested.

#### 2.1.2. 19-202(a)

**Figure 3:**
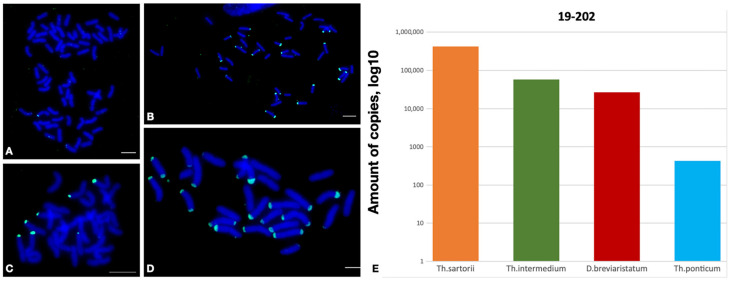
Localization of the 19-202 tandem repeat on the metaphase chromosomes of (**A**) *Thinopyrum ponticum* PI 636523; (**B**) *Thinopyrum intermedium* PI 401200; (**C**) *Dasypyrum breviaristatum* PI 516547; and (**D**) *Thinopyrum sartorii* PI 531745, using fluorescence in situ hybridization. Green signals indicate the chromosomal localization of 19-202 tandem repeat. The bar indicates 10 µm. (**E**) Histogram of the copy number of tandem repeat 19-202 in *Thinopyrum sartorii* PI 531745, *Thinopyrum intermedium* PI 401200, *Dasypyrum breviaristatum* PI 516547, and *Thinopyrum ponticum* PI 636523.

Previously, repeat 19-202 in the *Th. ponticum* (2*n* = 70, JJJJJJJ^s^J^s^J^s^J^s^) genome was identified, which was used for the comparative characterization of the wild relatives of wheat [28]. The qPCR results showed that numerous copies of this repeat were also present in other species of *Thinopyrum* with different ploidy levels, namely *Th. sartorii* (2*n* = 28, J^e^J^e^J^b^J^b^), *Th. intermedium* (2*n* = 42, J^r^J^r^J^vs^J^vs^StSt), and *D. breviaristatum* (2*n* = 28, VVVbVb). Notably, the copy number of repeat 19-202 in *D. breviaristatum* was found to be higher than that in *Th. ponticum,* with the latter showing the lowest copy number among the representatives of *Thinopyrum.* The results of the FISH analysis correlated with those of qPCR. Despite the fact that 19-202 was identified in the *Th. ponticum* genome, it was relatively low-copy, with the signals being bright and clear but localized to the smaller regions of the chromosomes. A significantly larger number of localization sites were observed in the chromosomes of the other *Thinopyrum* species and *D. breviaristatum* (Figure 3).

The presence of 19-202 in both the J and V genomes suggests that its expansion throughout the genomes began before their divergence in a common ancestor. However, 19-202 manifested variably in the different genomes, showing attenuation in the J-genome of *Th. ponticum* and amplification in the J and V genomes of the other *Thinopyrum* species and *D. breviaristatum*. All the species analyzed are polyploids with complex genomic constitutions. The localization of the repeat in the subterminal region, which is involved in chromosome segregation during meiosis, suggests that the copy number of this repeat may be related to the polyploidization of the species. Interestingly, the higher the ploidy level of the *Thinopyrum* species is, the lower the observed abundance of 19-202 will be, possibly due to its elimination at the time of polyploidization, which often occurs in the process of genome evolution [29]. Thus, the information on the copy number of the repeat obtained using qPCR is supplemented with information on the number and localization of the repeat hybridization sites on the chromosomes, enabling us to discuss its role in evolution and polyploidization. 

#### 2.1.3. CL244

**Figure 4:**
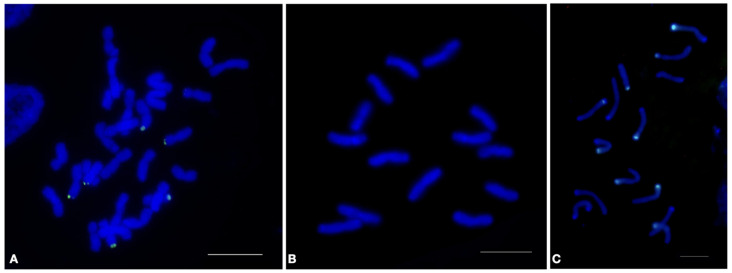
Localization of CL244 tandem repeat on metaphase chromosomes of (**A**)—*Aegilops crassa* AE 742; (**B**)—*Aegilops tauschii* K-112; (**C**)—*Thinopyrum bessarabicum* PI 201890, using fluorescence in situ hybridization. Green signals show chromosomal localization of CL244 tandem repeat, bar indicates 10 µm.

Repeat CL244 was identified in the genome of tetraploid *Aegilops crassa* (2*n* = 28, DDXX) consisting of a D subgenome, similar to the D genome of diploid *Aegilops tauschii* (2*n* = 14, DD) and an X subgenome of unknown origin, by analyzing the reads that did not align with the *Ae. tauschii* reference genome, i.e., by the application of a sequence filter [30]. Judging by the qPCR results, the copy number of CL244 in the genome of *Ae. tauschii* was lower than that of *Ae. crassa*, which corroborated the data obtained from the bioinformatical analysis. Simultaneously, the copy number of CL244 in *Th. bessarabicum* (2*n* = 14, JJ) was significantly higher than that of both *Aegilops* species (Figure S1). The results of the FISH analysis confirmed the conclusions drawn based on the qPCR results. Bright signals of CL244 in the *Ae. crassa* and *Th. bessarabicum* chromosomes and their absence in *Ae. tauschii* were observed (Figure 4).

The copy number of the CL244 repeat differed between the species with different ploidy levels. This may be explained by the fact that it exhibited a burst in the J genome, remained stable in the X genome, and was eliminated in the D genome following their divergence from the potential common ancestral genome. Using qPCR, the validity of the data of the bioinformatic analysis was confirmed, and it was ensured that the identified repeat met the requirements for conversion to a cytogenetic marker.

### 2.2. “Transfer of Repeats” Strategy for the Study of Closely Related Species

It is preferable to develop markers using whole-genome sequence (WGS) data obtained directly from the species for which the marker is being designed. However, in the absence of qualitative data from the whole-genome sequencing of the target species, it is possible to create markers based on repeats identified in the genomes of closely related species.

### 2.2.1. P720

**Figure 5:**
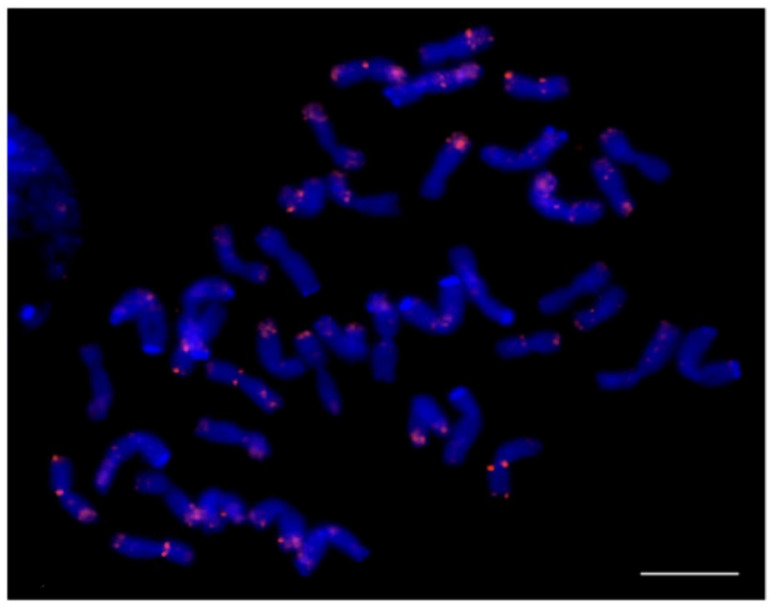
Localization of the P720 tandem repeat on the metaphase chromosomes of triticale cv. Solovey Kharkovskiy using fluorescence in situ hybridization. Red signals indicate the chromosomal localization of the P720 tandem repeat [25]. The bar indicates 10 µm.

*Ae. tauschii* (2*n* = 14, DD) was used as a donor to generate markers for triticale (2*n* = 42, AABBRR) [25]. The copy numbers of the *Ae. tauschii* repeats in *Triticum aestivum* (2*n* = 42, AABBDD), *Ae. tauschii*, and *Secale cereale* (2*n* = 14, RR) were estimated (Figure S2). Based on the qPCR results, P720 was selected as a probe for the FISH analysis, since the copy number of the P720 repeat is high in all these species (Figure 5).

The chromosome preparations of triticale show clear and bright signals of P720 in the chromosomes of all the subgenomes. Thus, although the origin of this tandem repeat is the D genome of *Ae. tauschii,* the results of the qPCR and FISH show a high copy number of the P720 repeat in the A, B, and R subgenomes of triticale, indicating its conservatism and antiquity.

### 2.2.2. 17-202

Wheat-wheatgrass amphidiploid hybrids (WWGHs) are important in breeding, enabling the transfer of valuable economic traits from wild cereals to cultivated wheat via the “breeding bridge.” The goal was to develop cytogenetic markers that are specific to wheatgrass chromosomes and suitable for application to wheat-wild relative hybrids. To exclude repeats that are abundant in the wheat genome, the copy numbers of tandem repeats identified in wheat and wheatgrass were compared [28].

Repeat 17-202, identified in the *Th. ponticum*, showed a high copy number in the genomes of the WWGHs, *Th. ponticum*, and *Th. intermedium* but a rather low copy number in *T. aestivum* (Figure S3). Since the aim was to develop markers specific to wheatgrass chromosomes, this tandem repeat was selected for FISH analysis.

**Figure 6:**
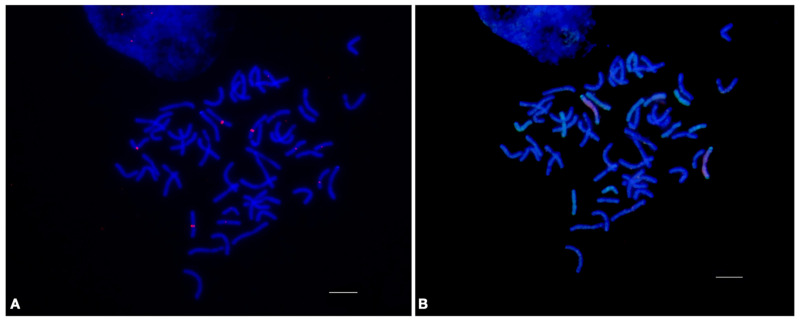
Localization of the 17-202 tandem repeat on the metaphase chromosomes of (**A**) wheat-wheatgrass hybrid 548 using fluorescence in situ hybridization and (**B**) genomic in situ hybridization (GISH) on the same chromosome spread hybridized to total genomic DNA of *P. spicata* (green) and *D. villosum* (red) as probes. Green signals indicate the chromosomal localization of the 17-202 tandem repeat. The bar indicates 10 µm.

To confirm the specificity of 17-202 to wheatgrass, a genomic in situ hybridization was performed using *D. villosum* (V genome) and *Pseudoroegneria spicata* (St genome) genomic DNA. GISH, with the labeled DNA of the V and St genomes, made it possible to distinguish between the different subgenomes of *Th. intermedium* [23,31,32]. Thus, it was shown that the signals of 17-202 belong to the wheatgrass chromosomes, since they hybridized with *D. villosum* and *P. spicata* DNA (Figure 6).

### 2.3. Analysis of Intraspecific Polymorphism

Often, the object of research is a population of individuals or several populations of the same species, two or more lines of crossing, representatives of the same species grown under different conditions, etc. In this case, qPCR should be used for the initial exploration of genetic diversity. The landscape of repeats can vary or be stable within a species, regardless of the geographical origin of its representatives. After a preliminary assessment of the TRs on various genotypes using qPCR, it is possible to select the markers of interest and convert them into cytogenetic markers.

#### 2.3.1. 17-62

**Figure 7:**
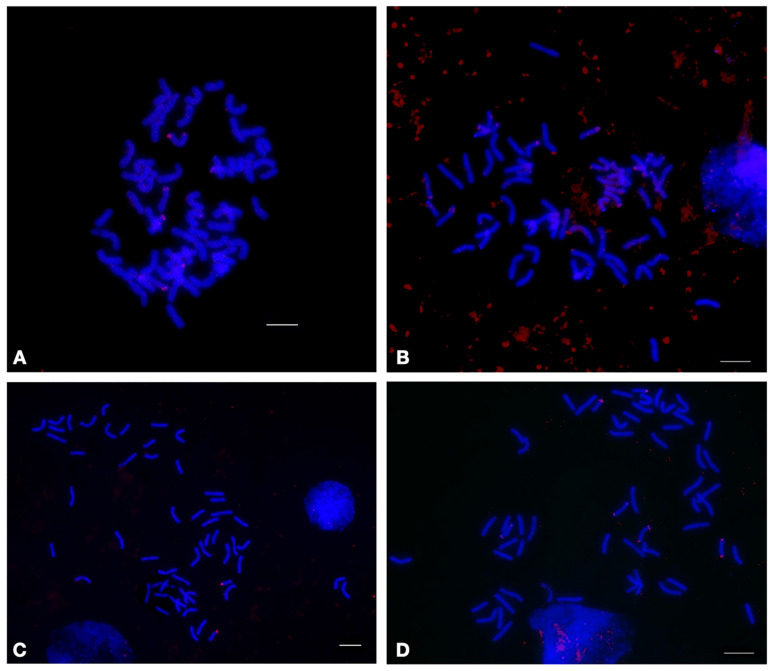
Localization of the 17-62 tandem repeat on the metaphase chromosomes of the wheat-wheatgrass hybrids (**A**) ZP26; (**B**) 166; (**C**) 4044; and (**D**) 548, using fluorescence in situ hybridization. Red signals indicate the chromosomal localization of the 17-202 tandem repeat. The bar indicates 10 µm.

Repeat 17-62 was identified in the *Th. ponticum* genome, with the aim of identifying wheatgrass chromosomes in the WWGHs (for further detail, see paragraphs 17-202 and [28]). The qPCR analysis showed that the copy number of this repeat in the genomes of *Th. intermedium*, *Th. ponticum*, and the WWGHs was significantly higher than that in the genome of common wheat, rendering it a good candidate marker of wheatgrass chromosomes (Figure S4). Moreover, its abundance correlated with the ploidy levels of the wheatgrass subgenomes: *Th. ponticum*, with five sets of wheatgrass chromosomes, exceeds *Th. intermedium*, with three sets and followed by the WWGHs, carrying only one set, and common wheat, with no wheatgrass chromosomes. Additionally, the copy number variation between the different WWGH accessions was noted, as visualized by FISH analysis (Figure 7).

Wheat-*Thinopyrum* amphidiploids combine a complete set of wheat chromosomes and part of the genome of polyploid wheatgrass (*Th. intermedium* and/or *Th. ponticum*). Meanwhile, each WWGH breeding line has its own combination of wheatgrass chromosomes, which determine the phenotypic characteristics and economically valuable traits. Thus, the reliable identification of specific wheatgrass chromosomes that are part of the WWGH genome and may carry valuable traits, such as resistance to stress factors or high quality, is an important task. Repeat 17-62, due to the detected differences in the copy number and FISH signal patterns, can be used to assess the diversity of WWGHs and as a PCR marker to monitor wheatgrass chromatin in introgression lines of wheat.

#### 2.3.2. 19-202(b)

The abovementioned repeat 19-202 was identified in the *Th. ponticum* genome and demonstrated a low abundance in this species [28]. qPCR studies on the *Th. ponticum* accessions of different geographical origins showed that the 19-202 copy number varied, and this was observable in FISH analysis.

**Figure 8:**
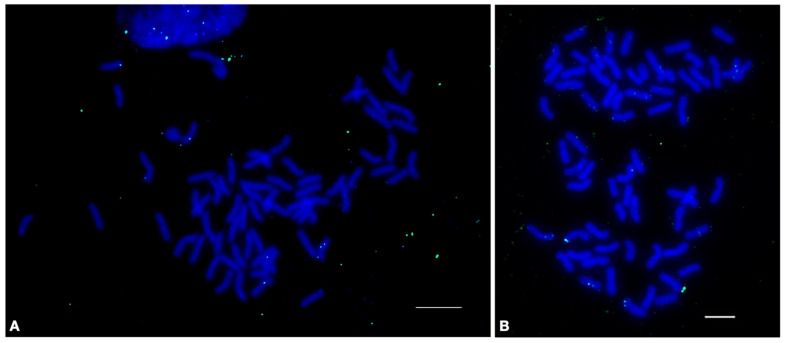
Localization of the 19-202 tandem repeat on the metaphase chromosomes of (**A**) *Thinopyrum ponticum* PI 693508 and (**B**) *Thinopyrum ponticum* 1158A/19 using fluorescence in situ hybridization. Green signals indicate the chromosomal localization of the 19-202 tandem repeat. The bar indicates 10 µm.

*Th. ponticum* accession 1158A/19 showed the highest relative copy number among the analyzed samples, followed by accessions PI 636523 and PI 547313, while the lowest abundance was demonstrated by accession PI 693508 (Figure S5). As indicated by FISH, 19-202 is localized terminally to the chromosomes of accession 1158A/19, with two small, clear signals being visible. No signals were detected in accession PI 693508 (Figure 8).

Repeat 19-202, which was identified in the study, can be used to study the population processes of *Th. ponticum*, assess diversity within and between populations, and estimate the influences of the growth conditions of certain populations on the repeatome. Thus, the repeats that show a constant copy number within one species that differs from the other species can be used as species-specific markers. Repeats that vary within a species can be used as a tool for population analysis.

### 2.4. Individual Cases

#### 2.4.1. HRTR12

DNA from male and female sea buckthorn (*Hippophae rhamnoides*) plants was used in this study. The HRTR12 repeat showed differences between the male and female plants according to the results of the bioinformatics analysis and was selected for further analysis using FISH [33].

The determination of the copy number was complicated by the lack of information regarding the reference single-copy gene in sea buckthorn. Thus, an approximate estimation of the copy number of HRTR12 was performed, based on the amplification cycle when the fluorescence level passed the threshold Cq. According to the qPCR results, the HRTR 12 repeat showed a lower copy number in the male plants compared to the female plants (Figure S6).

**Figure 9:**
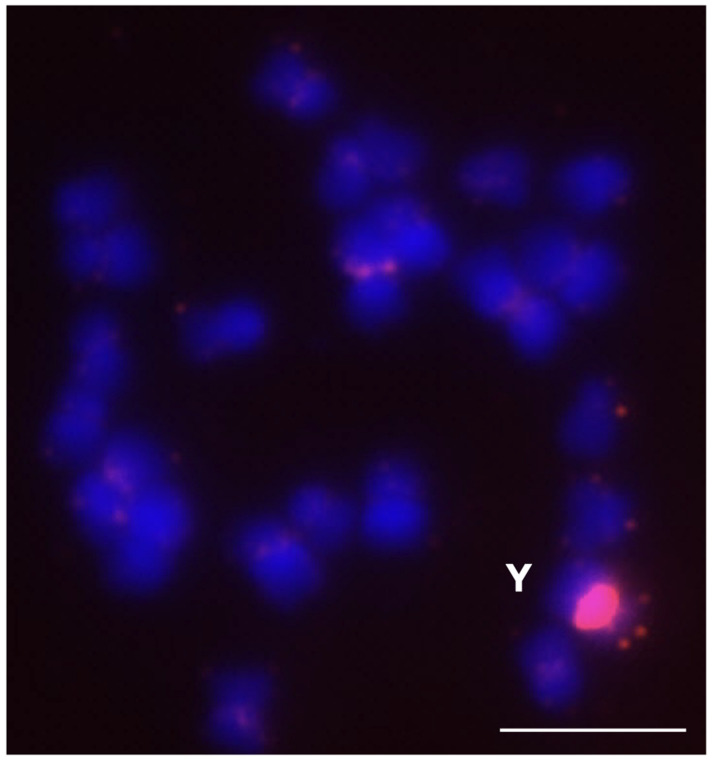
Localization of the HRTR12 tandem repeat on the metaphase chromosomes of *Hippophae rhamnoides* using fluorescence in situ hybridization. Red signal indicates the chromosomal localization of the HRTR12 tandem repeat. The letter “Y” corresponds to Y chromosome. The bar indicates 5 µm.

The FISH results showed the highest abundance of HRTR12 in the Y chromosome of the male plant. The signal was clear, while on the other chromosomes, the signals were weak and dispersed (Figure 9).

HRTR12, having been identified, showed a part of the sea buckthorn Y chromosome that differed from the X chromosome and probably does not participate in recombination with the latter during meiosis, providing additional information about the origin and evolution of the sex chromosomes of the plants. The example under consideration demonstrates the applicability of the algorithm, even in cases where there is no information about the reference single-copy gene, or when problems arise during the design of the primers.

#### 2.4.2. CL131

**Figure 10:**
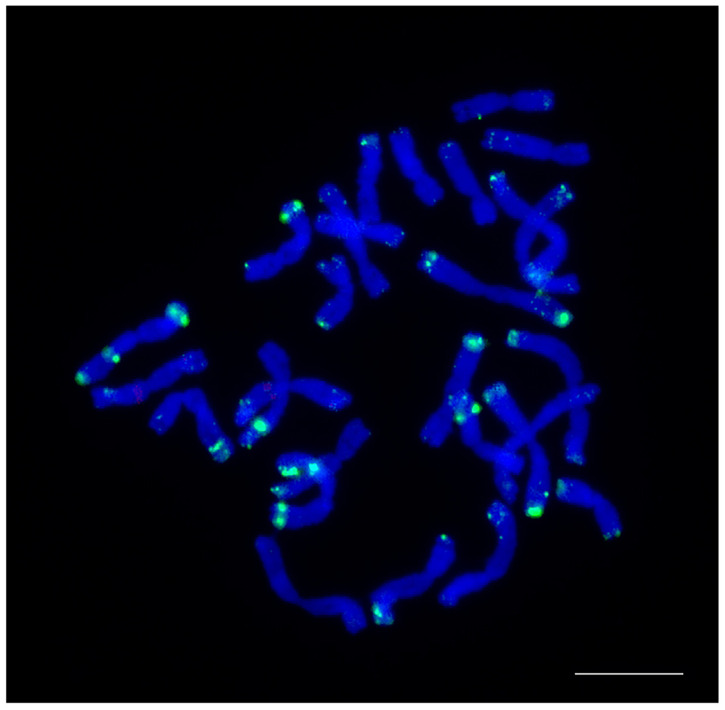
Localization of the CL131 tandem repeat on the *Aegilops crassa* AE 742 metaphase chromosomes using fluorescence in situ hybridization. Green signals indicate the chromosomal localization of the CL131 tandem repeat. The bar indicates 10 µm.

The CL131 repeat was identified in the *Ae. crassa* genome during the search for X-subgenome-specific repeats (see paragraph CL244, [30]). This repeat is a satellite repeat, and judging by the bioinformatical analysis, it has a relatively moderate copy number. However, the copy number revealed by qPCR was lower than expected, based on the in silico analysis (Figure S7) and compared with the other *Ae. crassa* repeats (see Figure S1). The FISH analysis indicated the sub-telomeric localization of CL131 in the *Ae. crassa* chromosomes and numerous bright and clear signals (Figure 10), which did not corroborate the qPCR results (Figure S7).

Most likely, such inconsistencies are due to the poor quality of the primers, which leads to the low efficiency of the reaction. To avoid such situations, it is recommended to determine the primer efficiency of the reaction by building a standard curve, and in cases of low performance, to design new primers.

#### 2.4.3. CL239

In the study of the *Ae. crassa* subgenome markers [30], a low-copy tandem repeat CL239 that showed promise as an *Ae. crassa*-specific repeat was identified. Judging by the bioinformatical data, this repeat consists almost entirely of the *Ae. crassa* reads and does not contain any reads of the closely related species *Ae. tauschii.* This indicates that it can hypothetically distinguish between *Ae. crassa* and *Ae. tauschii.*

When designing the primers for this repeat, the problem of self-complementation in the region of the 3’ ends of the primers was encountered. This led to the formation of primer dimers, which greatly distorted the qPCR results. The amplification curves exceeded the threshold Cq in the second PCR cycle, which completely contradicted the data obtained by bioinformatical analysis. Thus, it appears that the CL239 repeat is a low-copy tandem repeat (Figure S8a). The melting peak of the product was one at 71 degrees. Since the copy number was much higher than expected, primers without self-complementarity at the 3’ ends were redesigned. The copy number revealed by qPCR with the new primer pairs was close to the expected number (Figure S8b).

## 3. Discussion

In this article, several cases of the use of qPCR are considered, which can be categorized according to the objectives of the research, namely, the comparative analysis of closely related species, development of markers for one species using WGS data from another species, and the study of intraspecific polymorphism. The problems of the absence of a reference gene, discrepancies between the copy number estimates, and difficulties in the design of primers for repetitive sequences were also considered.

The comparison of two or more species of the same genus (paragraphs CL232, 19-202(a), CL244) demonstrated reproducible results and a high correlation between the number of copies of the repeat and the number of signals in the FISH analysis. A low copy number (10 copies or less) results in a lack of signals, as it is too low for the resolution of FISH. In other studies, data obtained by qPCR also corroborated the results of FISH when investigating the copy numbers of repetitive elements in both animals (*Drosophila melanogaster, Astatotilapia latifasciata, Eyprepocnemis plorans, Monopterus albus*) [20,21,22,24] and plant (*Ae. speltoides, Th. ponticum, Th. intermedium, T. aestivum,* triticale) species [7,14,25,28,34]. Moreover, the qPCR assay may supplement the FISH results with additional information. Thus, it demonstrated that the newly identified satellite repeat BnSAT200 is not associated with the CENH3 protein in *B. nigra* and enabled the direct comparison of the centromeric repeat abundance between the diploid progenitors of *Th. intermedium* [23,35]. 

When developing markers for one species using WGS data from another species, qPCR, as a preparatory step preceding FISH analysis, is necessary not only to assess the copy number and primer performance but also to address the question of whether or not this repeat occurs in the target accession, since there is no bioinformatic sequence analysis data to rely on, as demonstrated in the cases of P720 and 17-202. A similar study sought to distinguish the subgenomes of another allopolyploid, *Th. intermedium* (2*n* = 42, J^r^J^r^J^vs^J^vs^StSt), based on bioinformatic analysis and FISH without a qPCR assay [36].

The tandem repeats with varying abundance in different WWGHs (case 17-62) can be used as qPCR markers to assess the variability and diversity of the *Thinopyrum* component of WWGHs or other intergeneric hybrids of wheat in cases where alien chromatin should be assessed. Compared to karyotyping using GISH and FISH, qPCR profiling using primers for the variable *Thinopyrum*-specific tandem repeats enables the rapid identification of the most genetically different and distant forms in a wide range of breeding lines. The most genetically different lines are then subjected to phenotyping, either in multiple-year field experiments or in the laboratory using high-throughput digital phenotyping systems, together with the modeling of drought or other stresses. Such an approach aids in the search for correlations between the variations in the *Thinopyrum* (or other wheat-related species) components and valuable agronomic traits, such as resistance to biotic and abiotic stresses. The same assay can be applied to the preliminary estimation of wild-wheat-related species in pre-breeding. When phenotyping under stress conditions, it may be useful to select the accessions with the most different qPCR profiles (case 19-202(b)).

The study of intraspecific polymorphism (paragraphs 17-62 and 19-202(b)) can be performed through a pre-assessment of the qPCR results. The qPCR method has been widely used to study the dynamics of mobile elements on the evolutionary scale [15] and breeding scale [13] following hybridization or self-pollination [16,17], different timescales of polyploidization [37], and the dynamics of environmental influences [18]. New satellite repeats identified by qPCR that display differences between accessions can also be used to study genome dynamics over different timescales. Studying the variability in the quantitative composition of satellites is proposed. Previously, qPCR applied to repeats as Spelt1 [14,34], Spelt52, and 5S rDNA [38] was used for the comparative analysis of populations. Expanding the pool of known satellite repeats through new strategies for their identification, using a broader range of bioinformatic approaches and WGS, is proposed.

The estimation of the copy-number of a repeat by qPCR can be carried out by normalization to a single-copy gene [14,20,23] or by comparison with samples with known copy numbers [12,38]. However, information regarding the presence of true single-copy genes in a given species may be scarce, while the preparation of a library with a known concentration is an additional step. This problem was avoided in the study (paragraph HRTR12) by comparing the Cq of the accessions. The optimization of the qPCR protocol itself improves the informative value of the results. For example, the differences between high-copy repeats will be more pronounced when low concentrations of DNA are used in the reaction. In contrast to the concentration, based on the authors’ experience and other studies, the size of the qPCR amplicon for copy number estimation may vary within a wide range (65 bp in [21], 218–432 bp in [22], 369–4300 bp in [38]). For similar tasks, Taqman probes can be used [21]. However, dyes such as EvaGreen, SYBRGreen, and their analogs are much easier and cheaper to apply.

High-throughput sequencing methods are becoming more accessible, and the amount of information to be processed is growing. The search for tandem repeats has become a routine task, and the focus of researchers is shifting toward the selection of potential molecular markers based on their abundance. In addition to molecular genetic selection methods (qPCR), one can turn to bioinformatic approaches, including the selection of repeats based on their homology with known repeats and sequence filtering before the application of the RepeatExplorer2 pipeline (paragraph CL244).

FISH is a popular method of analysis; however, due to its laboriousness, it requires the preliminary selection of markers that are practically, not just theoretically, suitable for the laboratory work. Classical PCR only provides a qualitative, binary result regarding the absence or presence of the tandem repeat of interest in the genome, and this information is not enough. qPCR provides more complete, quantitative information in a shorter time. The seamless transition from a qPCR study to cytogenetics study is also ensured by the ease of converting a PCR product into a FISH probe ([23,35]; this study). Using the same primers for these purposes is suggested, whereas in many similar studies, different primers were used for qPCR and FISH, respectively [20,38].

Previously, the potential of qPCR for marker development based on the donor species for the target species was demonstrated [25]. In this article, a wider range of studies in which qPCR was used to pursue different goals are discussed. However, the strategy remained consistent, comprising the search for tandem repeats followed by qPCR analysis and the conversion of the selected tandem repeats into cytogenetic markers.

The developed chromosomal markers are useful for the identification of individual chromosomes by FISH, and combined with genomic data, they can be further employed in both evolutionary and applied research. The precisely localized chromosomal markers provide basic information for comparisons between homologous chromosomes or chromosomes involved in sex determination. They can be effectively used as a tool to monitor translocations between chromosomes in introgressive lines obtained via wide hybridization. In this case, for example, CL244 was successfully localized to 1XcrL, AcrL, and 4D1S in tetraploid *Ae. crassa* and 1JS, 3JL, 5JS, 6JS, and 7JS in *Th. bessarabicum* [30]. Meanwhile, HRTR12 mainly hybridized to the sea buckthorn Y chromosome [33], while 17-202 was specifically localized to wheatgrass chromosomes, thus differentiating them from wheat chromosomes [28].

## 4. Materials and Methods

### 4.1. Plant Materials

The studied plant accessions and the particular case in which they are considered are shown in Table 1.

### 4.2. Methods

In general, the overall procedure in the research consists of the following steps:

1. Finding repeats with RE2 and/or TRfinder
2. qPCR with found tandem repeats
3. FISH with selected tandem repeats

#### 4.2.1. DNA Extraction 

The genomic DNA was extracted from the leaves of growing plants, as described in [39] in cases pHv-961, 19-202, 17-62, 17-202, CL244, CL131, CL239, P720, and in [40] in case HRTR12, and was used for whole-genome dried sequencing, qPCR, and probe preparation for FISH. The quality and quantity of the extracted DNA were checked using a NanoDrop OneC spectrophotometer (Thermo Fisher Scientific, Madison, WI, USA).

#### 4.2.2. Sequencing and Preprocessing

Whole-genome libraries were carried according to Swift 2S Turbo protocol (Swift Bioscience, Ann Arbor, MI, USA) in cases 19-202, 17-62, 17-202, CL244, CL131, CL239; amount of DNA was 25 ng. The run was performed with Illumina protocols on the Illumina NextSeq with NextSeq 500/550 Mid Output Kit v2.5 (300 cycles) (Illumina, San Diego, CA, USA) with pair-end reads. The length of read was 151 bp; the length of index was 8 bp. Illumina sequencing was ordered in Genomed, Ltd. (Moscow, Russia) in cases 19-202, 17-62, 17-202, CL244, CL131, CL239. One Illumina MiSeq sequencing run was performed for each male and female genomic DNA in case HRTR12. 

Sequencing reads were analyzed by quality control tool FastQC v0.11.5, followed by quality filtering based on the sequence quality score, adaptors trimming, filtering reads shorter than 100 bp (in the cases 19-202, 17-62, 17-202), trimmed to length 230 bp (in the case of HRTR12), or unpaireds using the Trimmomatic v0.39 sequence tool. Quality-filtered reads were randomly sampled to 10,000,000 paired-end reads, of which 4,752,527 were analyzed using the RepeatExplorer2 pipeline [41] (in cases 19-202, 17-62, 17-202) and 415,650 paired-end reads for both male and female individuals and the reads were merged together (in the case of HRTR12. In cases CL244, CL131, and CL239, adapter sequence and low-quality reads were removed by bbduk.sh from BBMap package (v38.90). After that, they were trimmed from the 3′-end, in order that all the reads prepared for assembly were the same fixed length. We have controlled the quality of resulting reads by using FastQC, and sampled 2,000,000 paired interlaced reads from them. Interlaced reads were then forwarded to RepeatExplorer2.

#### 4.2.3. Repeat Search

RepeatExplorer2 pipeline was used for repeat searching in cases 19-202, 17-62, 17-202, CL244, CL131, CL239, HRTR12. In case CL244, trimmed reads *Ae. crassa* were mapped on *Ae. tauschii* Aet v4.0 genome assembly using bwa mem v0.7.17. For further analysis, reads perfectly mapped on reference assembly were removed using samtools [42]. The resulting 831,000 reads were used for de novo tandem repeats identification using RepeatExplorer2 pipeline. The *Ae. tauschii* genome contigs, available at the National Center for Biotechnology Information NCBI (MCGU01000001–MCGU01068537), were used for the TR search using Tandem Repeats Finder (TRF) software Version 4.09 (Boston University, Boston, MA, USA) in case P720. In the case pHv-961 Hordeum vulgare, pHv-961 repeat (HM536205.1) was found at National Center for Biotechnology Information NCBI.

#### 4.2.4. qPCR

Primers for repeat monomers were designed using Primer3 v0.4.0 software (Supplementary Table S1). Real-time qPCR, using primers developed for repeat monomers, was performed in triple technical replication, with water as negative control and VRN1 as a reference gene [37] according to the protocol described in [7]. The amplification was performed using CFX Real-Time PCR Detection System (Bio-Rad, Hercules, CA, USA) and Eva Green qPCR master mix (Syntol Ltd., Moscow, Russia) according to the manufacturer’s protocol. Primers were synthesized at Syntol Ltd. (Moscow, Russia). The primer concentration was 10 ng/μL, and the DNA concentration was 0.4 ng/μL. The amplification program was as follows: pre-incubation for 10 min at 95 °C, then 45 cycles: denaturation for 10 s at 95 °C; primer annealing for 30 s at 60 °C. The relative quantity (RQ) was calculated using Bio-Rad CFX Manager 3.1 software based on the obtained Cq volumes (in cases 19-202, 17-62, 17-202, CL244, CL131, CL239). 

In cases HRTR12, P720, and pHv-961, qPCR was performed using the following protocol: preincubation in 95 °C for 10 min; then 40 cycles: denaturation for 10 s at 95 °C; primer annealing for 30 s at 60 °C. The primer concentration was 10 ng/μL, and the DNA concentration was 0.4 ng/μL. We used three male and three female plants as DNA templates, each experiment was repeated independently twice. The qPCR amplification was run on the LightCycler^®^ 96 instrument (Roche Diagnostics, Mannheim, Germany). Each reaction was performed in a 15 μL volume consisting of 6 μL of reaction mix containing Eva Green ^®^ (Syntol Ltd., Moscow, Russia).

Statistical analysis, including calculating mean Cq values, standard deviation (Supplementary Tables S2–S11), and related copy-number against VRN1 reference gene (Figure 1 and Figure 2, Figures S1–S5, S7), was performed using Bio-Rad CFX Manager 3.1 software and Lightcycler 96 Software 1.1 (Roche, Basel, Switzerland).

#### 4.2.5. FISH Protocols 

Chromosome spread preparations were made from root tip using the squashing technique, as described in [43,44], in all cases except for HRTR12, where the steam-drop technique was applied, as described in [33,45]. 

Probes for FISH were generated from the PCR amplicons produced using the same primers as were used for the qPCR assay (Supplementary Table S1). PCR amplification was performed in a 15 μL reaction mixture containing approximately 50 ng genomic DNA, 1.5 μL of 10 × PCR buffer, 1.5 mM MgCl 2, 0.2 mM of dNTPs, 0.3 μM of each primer (synthesized by Syntol Ltd., Moscow, Russia), and 0.5 unit of Taq DNA polymerase. The PCR conditions were as follows: an initial denaturation step of 95 °C for 5 min, followed by 30 cycles of 94 °C for 1 min, annealing at 60 °C for 1 min and elongation at 72 °C for 1 min, with a final extension step at 72 °C for 5 min. The obtained amplicons were labeled by PCR according to the manufacturer’ s instructions with either biotin-16-dUTP PCR labeling mix (cases pHv-961, P720, 17-202, and 17-62), digoxigenin-11-dUTP PCR labeling mix (cases 19-202, CL244, and CL131) (Roche Molecular Biochemicals, Germany) or biotin-11-dUTP 1/3 PCR labeling Mix (case HRTR12) (Syntol Ltd., Moscow, Russia). In case 17-202, the probes for GISH were the *P. spicata* (St genome) and *D. villosum* (V genome) genomic DNA (50 ng/preparation), labeled with digoxigenin-11-dUTP and with biotin-16-dUTP, respectively, by nick translation, according to the manufacturer’s instructions (Roche, Germany).

FISH was carried out following the procedure described in [46] in all cases, except for case HRTR12, where protocol [47] was performed. In case 17-202, sequential GISH was carried out as described in [23]. After hybridization, the chromosomes were counterstained with 1 mg/mL DAPI. The detection was performed using streptavidin-conjugated Cy3 or FITC (Roche, Basel, Switzerland). Signals were visualized and recorded using an AxioZeiss Imager M1 fluorescence microscope eqipped with AxioCam MRm CCD camera (Carl-Zeiss, Oberkochen, Germany) in all cases, except for CL131, CL244, and CL239 where Leica DM6 B epifluorescence microscope equipped with DFC 9000 GTC camera (Leica, Wetzlar, Germany) was used.

## 5. Conclusions

1. qPCR can be used as a downsampling tool for satellite repeats that are most promising for use as chromosomal markers. This approach makes it possible to develop chromosomal markers for one species based on whole-genome sequences of a related species.
2. qPCR can detect tandem satellite repeats with several orders of magnitude difference in copy number between species, visualized by FISH. A correlation was shown between the results of bioinformatic analysis, qPCR, and FISH in most of the cases considered. In case of inconsistency in trends between qPCR and FISH results, we recommend checking the qPCR primers and designing other primers if necessary.
3. Primers designed for the satellite repeat monomer can be used for both qPCR and probe preparation for the FISH procedure.
4. Detected differences in qPCR copy number of satellite repeats allows for comparative analysis between closely related species, different populations of the same species, distant hybrids, and dioecious plants.

## Figures and Tables

**Table 1:** Plant material.

Species	Accession	Purpose (qPCR/FISH/Sequencing)	Case (Paragraph)
*Ae. crassa*	K-2485	qPCR, FISH	CL239
*Ae. crassa*	AE 742	qPCR, FISH, sequencing	CL244, CL131, CL239
*Ae. crassa*	AE 1649	qPCR, FISH	CL239
*Ae. tauschii*	K-112	qPCR, FISH, sequencing	CL244, CL131, CL239
*Ae. tauschii*	Clae 3	qPCR	P720
*D. breviaristatum*	PI 516547	qPCR, FISH	pHv-961, 19-202(a)
*D. villosum*	PI 21717	qPCR, FISH	pHv-961
*H. rhamnoides*	Botanicheskaya lyubitelskaya	FISH, sequencing	HRTR12
*H. rhamnoides*	Darlovo 7 (Male)	qPCR	HRTR12
*H. rhamnoides*	Darlovo 10 (Female)	qPCR	HRTR12
*H. rhamnoides*	Krasnoyarsk 3 (Female)	qPCR	HRTR12
*H. rhamnoides*	Moshkovo 2 (Male)	qPCR	HRTR12
*H. rhamnoides*	Dolzhanskaya 11 (Male)	qPCR	HRTR12
*H. rhamnoides*	Dolzhanskaya 18 (Female)	qPCR	HRTR12
*H. rhamnoides*	Pollinator 1	FISH, sequencing	HRTR12
*S. cereale*	EM1	qPCR	P720
*T. aestivum*	Chinese Spring	qPCR, FISH	CL244
*T. aestivum*	Ivolga	qPCR	P720
*T. aestivum*	Chinese Spring	qPCR, FISH	CL131, CL239
*T. aestivum*	Nemchinovskaya 56	qPCR	17-202, 17-62
*Th. bessarabicum*	PI 201890	qPCR, FISH, sequencing	CL244, CL239
*Th. intermedium*	PI 401200	qPCR, FISH	19-202(a), 17-202, 17-62
*Th. ponticum*	PI 693508	qPCR, FISH	19-202(b)
*Th. ponticum*	1158A/19	qPCR, FISH, sequencing	19-202(b)
*Th. ponticum*	PI 547313	qPCR, FISH	19-202(b)
*Th. ponticum*	PI 636523	qPCR, FISH, sequencing	19-202(a), 19-202(b), 17-202, 17-62
*Th. sartorii*	PI 531745	qPCR, FISH	19-202(a)
*Triticosecale*	Solovey Kharkovskiy	FISH	P720
WWGH	ZP26	qPCR, FISH	17-62
WWGH	166	qPCR, FISH	17-62
WWGH	548	qPCR, FISH	17-202, 17-62
WWGH	4044	qPCR, FISH	17-62

## Data Availability

Data is contained within the article and supplementary material.

## Supplementary Materials

The following supporting information can be downloaded at: https://www.mdpi.com/article/10.3390/plants12010080/s1, Supplementary Figures S1–S8 and Supplementary Tables S1–S11. Supplementary Figures S1–S5 and S7 show histograms of copy number, while Supplementary Figures S6 and S8 show amplification curves of particular tandem repeats in the studied species. Supplementary Table S1 show primers designed for monomers of satellite repeats, Supplementary Table S2–S11 show Cq mean and Cq standard deviation values for particular tandem repeats in the studied species.
