# Supplementary material for: qPCR as a Selective Tool for Cytogenetics
Source: Plants (Basel). 2022 Dec 23;12(1):80. doi: 10.3390/plants12010080 (PMC9824742; doi:10.3390/plants12010080)
Supplement: Supplementary file 1 [file plants-12-00080-s001.zip › Supplementary Figures S1-S8.pdf]

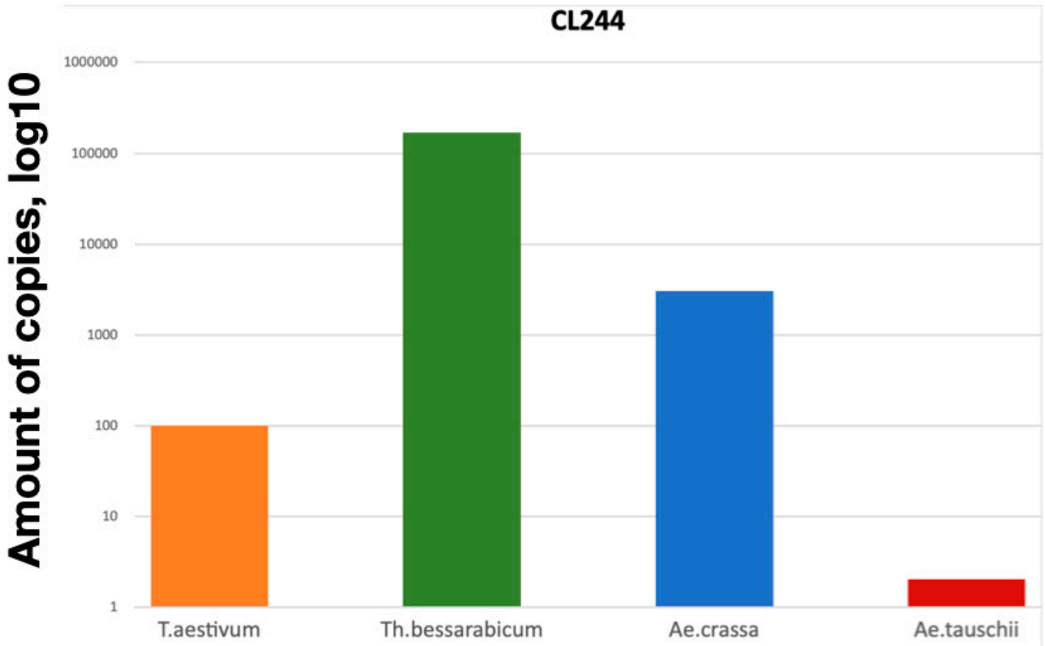

Figure S1. Histogram of copy number of the CL244 tandem repeat in *Triticum aestivum, Thinopyrum bessarabicum* PI 201890*, Aegilops crassa* AE 742*, Aegilops tauschii* K-112

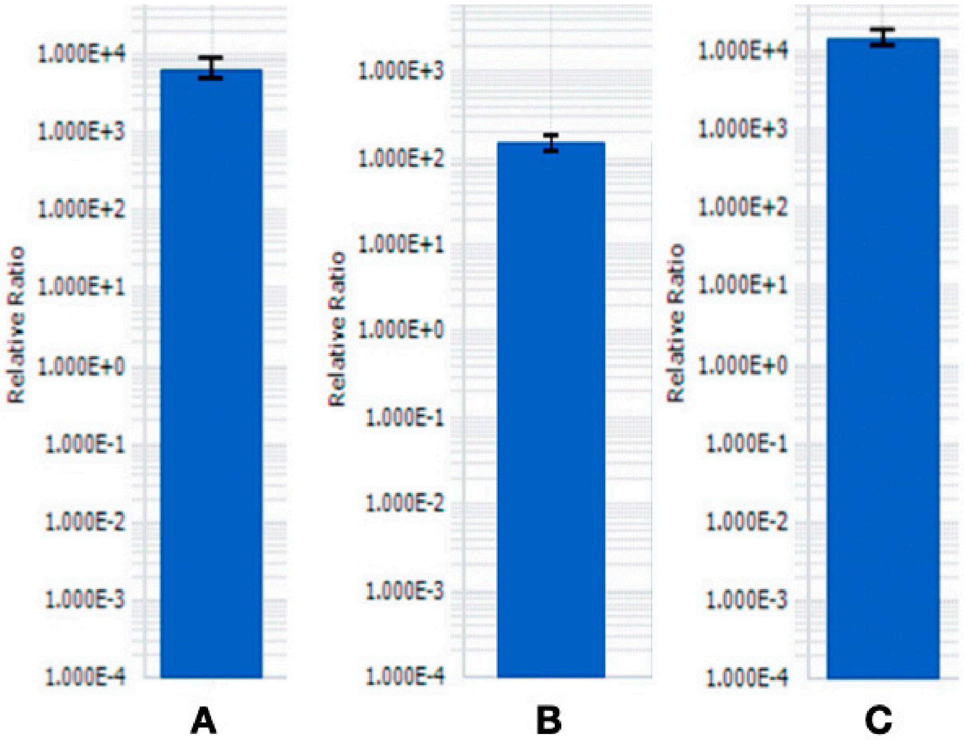

i

Figure S2. Histogram of copy number of the P720 tandem repeat in (A) - *Triticum aestivum* cv Ivolga; (B) - *Secale cereale* EM1; (C) - *Aegilops tauschii* Clae 3

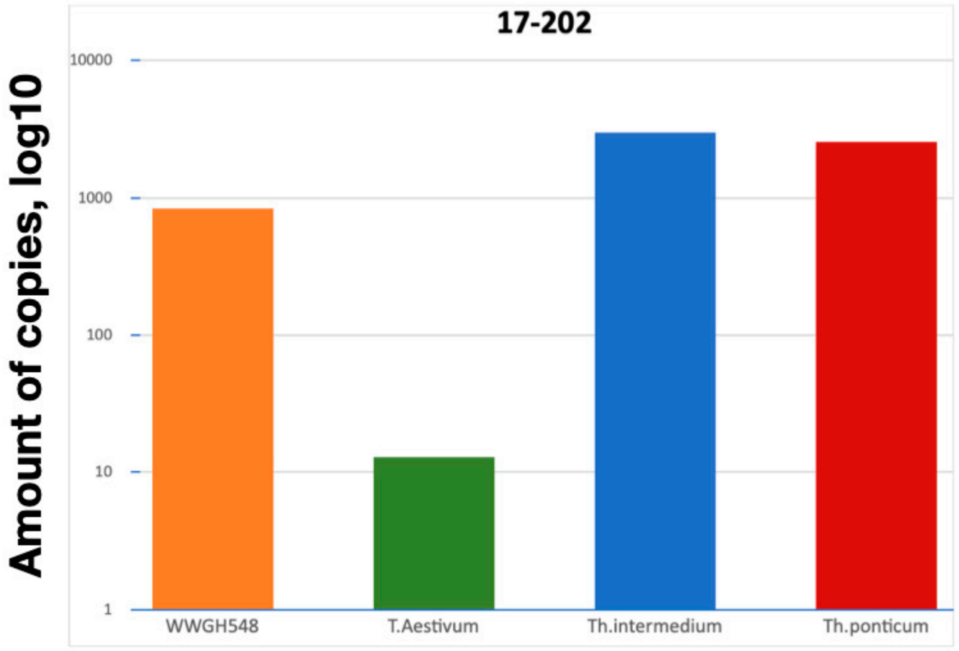

Figure S3. Histogram of copy number of the 17-202 tandem repeat in wheat-wheatgrass hybrid 548, *Triticum aestivum cv* Nemchinovskaya 56, *Thinopyrum intermedium* PI 401200, *Thinopyrum ponticum* PI 636523

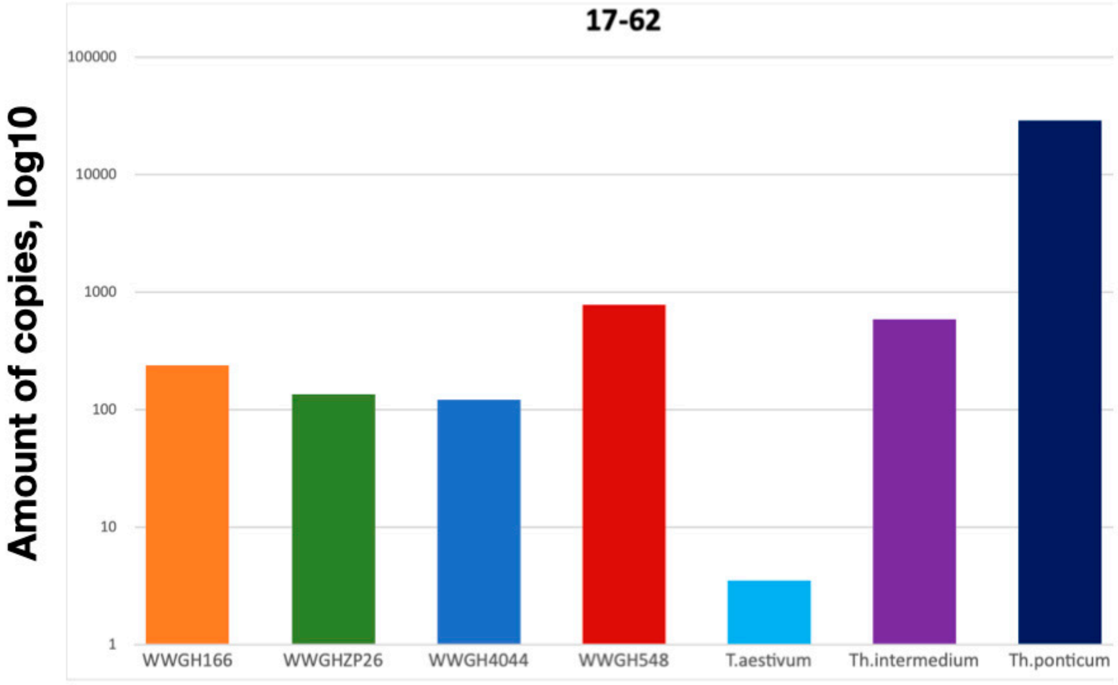

Figure S4. Histogram of copy number of the 17-62 tandem repeat in wheat-wheatgrass hybrids, *Triticum aestivum cv* Nemchinovskaya 56, *Thinopyrum intermedium* PI 401200, *Thinopyrum ponticum* PI 636523

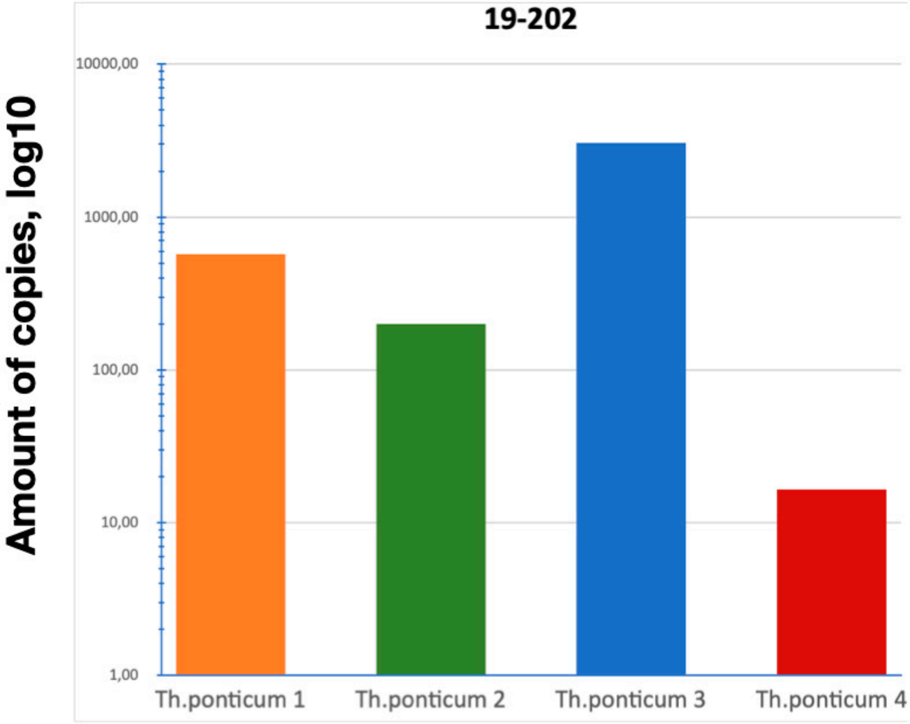

Figure S5. Histogram of copy number of the 19-202 tandem repeat in four different *Thinopyrum ponticum* accessions: *Th.ponticum* 1 - PI 636523; *Th.ponticum* 2 - PI 547313; *Th.ponticum* 3 - 1158A/19; *Th.ponticum* 4 - PI 693508

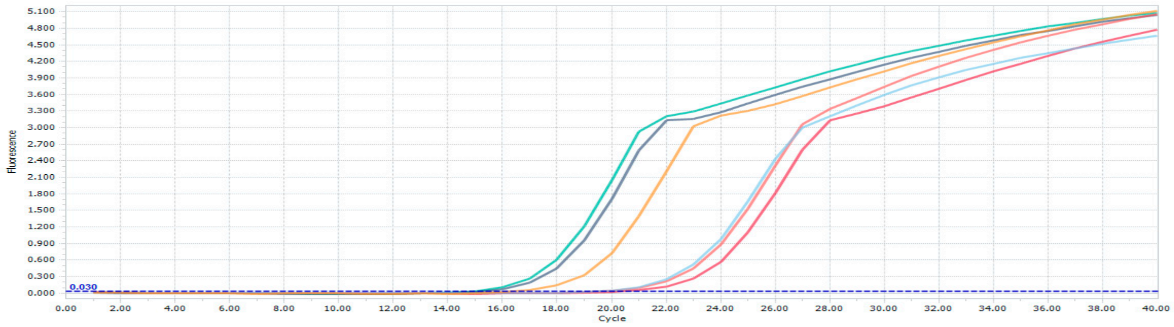

Figure S6. Amplification curves of the HRTR12 tandem repeat in the male (16-18 Cq) and female (20-22 Cq) sea buckthorn plants

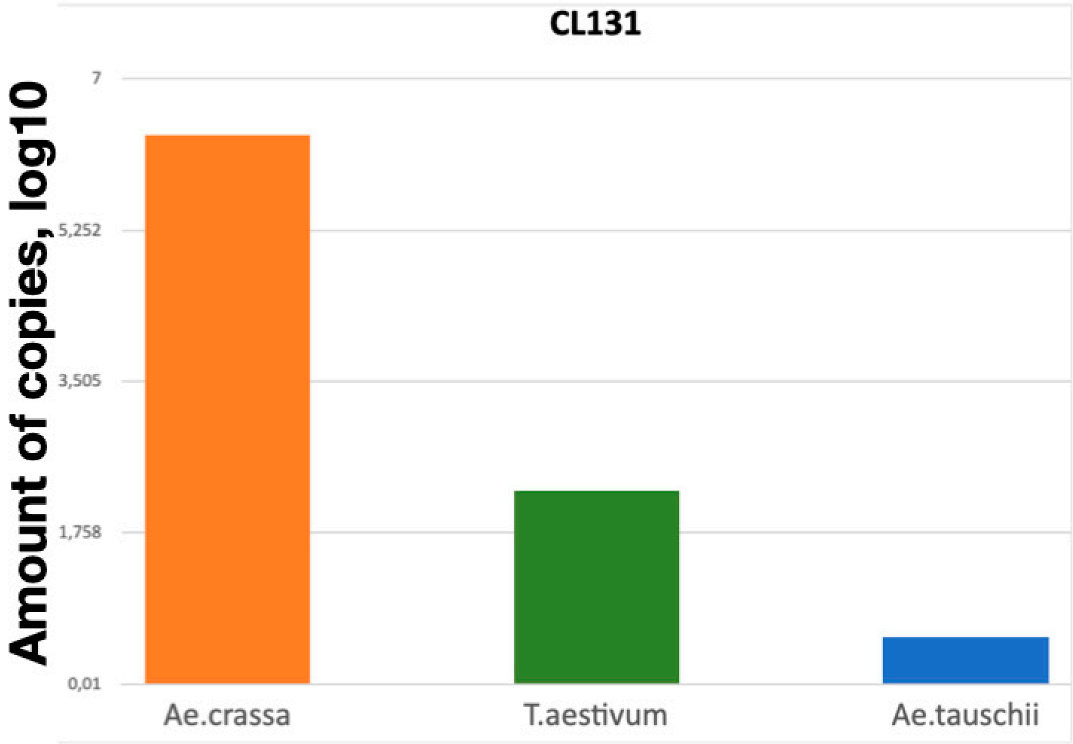

Figure S7. Histogram of copy number of the CL31 tandem repeat in *Aegilops crassa* AE 742, *Triticum aestivum cv* Chinese spring, *Aegilops tauschii* K-112

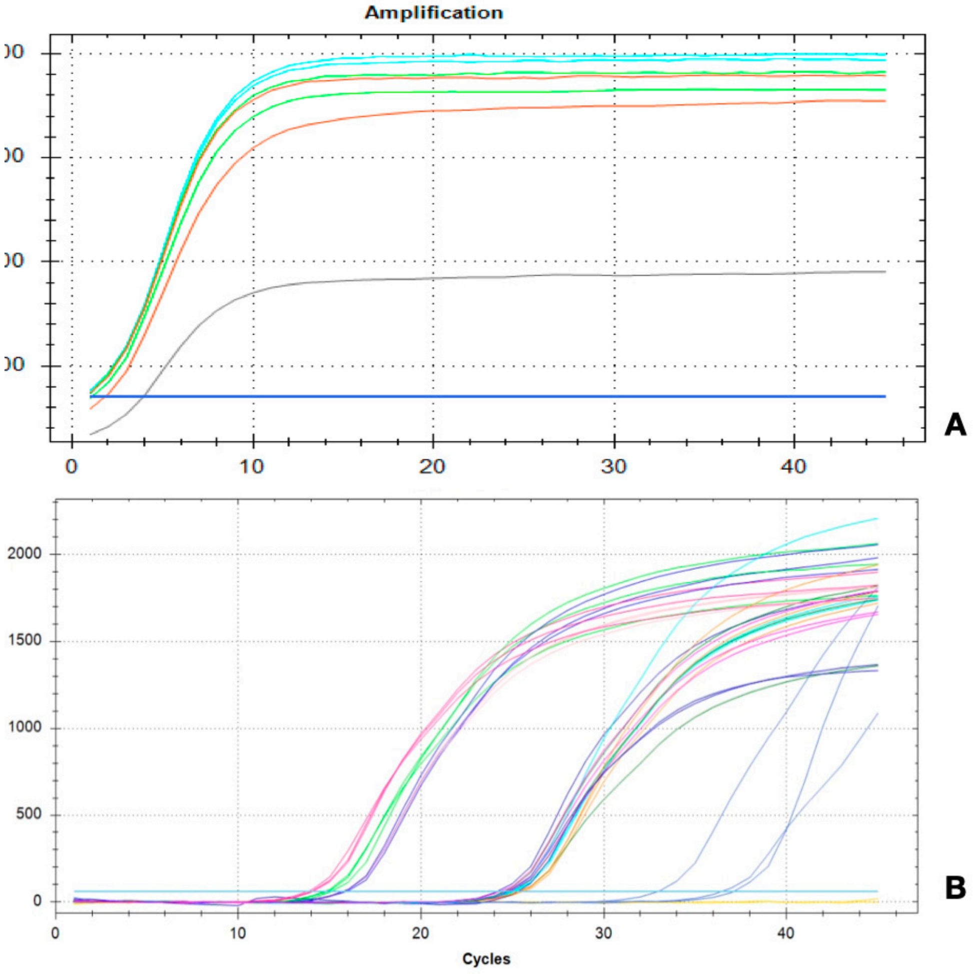

Figure S8. Amplification curves of the CL239 tandem repeat, (A) - primers with a high 3'-complementarity score; (B) - primers without 3'-complementarity.
